# Responses to language barriers in consultations with refugees and asylum seekers: a telephone survey of Irish general practitioners

**DOI:** 10.1186/1471-2296-9-68

**Published:** 2008-12-22

**Authors:** Anne MacFarlane, Liam G Glynn, Phillip I Mosinkie, Andrew W Murphy

**Affiliations:** 1 Department of General Practice, National University of Ireland, Galway, Ireland

## Abstract

**Background:**

Refugees and asylum seekers experience language barriers in general practice. Qualitative studies have found that responses to language barriers in general practice are *ad hoc *with use of both professional interpreters and informal interpreters (patients' relatives or friends). However, the scale of the issues involved is unknown. This study quantifies the need for language assistance in general practice consultations and examines the experience of, and satisfaction with, methods of language assistance utilized.

**Methods:**

Data were collected by telephone survey with general practitioners in a regional health authority in Ireland between July-August 2004. Each respondent was asked a series of questions about consulting with refugees and asylum seekers, the need for language assistance and the kind of language assistance used.

**Results:**

There was a 70% (n = 56/80) response rate to the telephone survey. The majority of respondents (77%) said that they had experienced consultations with refugees and asylum seekers in which language assistance was required. Despite this, general practitioners in the majority of cases managed without an interpreter or used informal methods of interpretation. In fact, when given a choice general practitioners would more often choose informal over professional methods of interpretation despite the fact that confidentiality was a significant concern.

**Conclusion:**

The need for language assistance in consultations with refugees and asylum seekers in Irish general practice is high. General practitioners rely on informal responses. It is necessary to improve knowledge about the organisational contexts that shape general practitioners responses. We also recommend dialogue between general practitioners, patients and interpreters about the relative merits of informal and professional methods of interpretation so that general practitioners' choices are responsive to the needs of patients with limited English.

## Background

Refugees and asylum seekers have complex health and social care needs due to the experiences that lead to forced migration as well as the experience of seeking asylum itself [1-4]. Research about their healthcare in host countries is important and the scope for improvement in general practice has been noted in European countries [5-8].

Language is one major barrier for refugees and asylum seekers in general practice. This matters because patients with limited English are less likely to engender empathic response from doctors, establish rapport in these relationships, receive sufficient information about their health or participate in decision making [9]. This barrier is shared by refugees and asylum seekers with other migrants but, arguably, refugees and asylum seekers have a specific, complexity to their health and social care needs [10,11] which means that their inability to communicate full details of their medical and social history can have specific, negative implications. For instance, in Ireland, general practitioners complete medico-legal records for asylum seekers for their applications for refugee status. An asylum seeker who cannot fully communicate a history of trauma or abuse to a general practitioner may not have a comprehensive medico-legal record and may be unsuccessful in their application for refugee status as a result.

Health policies advocate the use of trained, professional interpreters to address language differences and resultant communication/relationship difficulties [12-14]. A recent study found that the provision of trained, professional interpreters in general practice in the United Kingdom was generally good [7]. However, the bulk of research in this area indicates that the uptake of trained, professional interpreters in practice is *ad hoc *[15-20]. Service provider's knowledge of available services is patchy and there is reliance on informal interpreters (patients' children, relatives and friends) as a pragmatic response to acute consultation needs. Sometimes, no interpreter is used at all and there is an attempt to 'get by' with gestures and body language [15,18]

These findings are from qualitative studies and provide important accounts of refugees, asylum seekers and general practitioners' experiences and the contexts in which these occur. However, there is a dearth of quantitative data about *how often *language assistance is required in general practice, the *scale *of general practitioners knowledge about professional interpreting services, *how frequently *the various interpretation methods are utilised and the perceived merits and demerits of these among general practitioners. The present study was designed to address this knowledge gap.

## Methods

### Context: Refugees and asylum seekers in Ireland

Ireland is experiencing unprecedented inward migration [21]. Refugees and asylum seekers account for some of this pattern. There were 4,626 applications for declaration as a refugee in 1998. This reached a peak in 2002 with 11,634 applications. A decline is evident since with 4006 applications made in 2006 [22]. A dispersal policy is in operation since 2000 and asylum seekers are accommodated in direct provision centres around the country. As of June 20^th ^2008, there are direct provision centres in 22 of 26 counties in the Republic of Ireland with a total population of 8,157 asylum seekers .

Asylum seekers are frequent attenders in Irish general practice, attending twice as much as their Irish counterparts [4]. There is no statutory interpreting service for healthcare services. The Health Service Executive has contracts with commercial interpreting services. These usually operate with untrained interpreters [23]. In Ireland, the term 'professional interpreter' refers to status as a paid employee rather than professional membership.

### Area under study

The area under study was the Health Services Executive Western Area counties Galway, Mayo and Roscommonn (HSE WA). This area has a mixed urban and rural population of 326,500 people and has 228 GMS registered general practitioners. It has been receiving asylum seekers since 2000 and has a number of direct provision centres accommodating refugees and asylum seekers.

### Design

A list of active general practitioners currently treating refugees and asylum seekers was acquired from the Primary Care Department of the HSE WA. A letter was sent to all of the above notifying them of the study and this was followed up with telephone contact during which they were given the opportunity to partake in the study. Up to four attempts were made to make telephone contact with each study participant. A telephone survey was chosen over postal survey because it is known that response rates to postal surveys by general practitioners are often low [24].

A questionnaire was developed to establish respondents' experiences of the language barrier: the need for language assistance, their knowledge and use of professional interpreters and use of informal interpreters (summary in Additional file 1). The full questionnaire (shown on ) consisted of a list of twenty three questions, 11 of which were closed and 12 of which were open ended questions. This questionnaire was developed after extensive review of the international literature and consultation with experts in refugee and asylum seeker health, language interpretation and primary care. It was piloted with general practitioners in a neighbouring county who were not going to be part of the main study. The questionnaire was administered by PM under the supervision of AMacF. Administration of the questionnaire took between 10–15 minutes. Ethical approval was obtained from the research ethics committee of the Irish College of General Practitioners.

### Statistical methods

The data were inputted into SPSS (Windows: version 11.0) for analysis and double-checked. For respondents and non-respondents comparisons of mean age were made using independent-samples t test while comparisons of gender and GMS status were made using Χ^2 ^analysis.

## Results

### Study population

There were 80 general practitioners in the study area at the time of data collection actively treating refugees and asylum seekers. The response rate of the telephone survey was 70% (56/80). There was no significant difference between respondents and non-respondents in terms of gender and age (Table 1).

**Table 1:** Comparison between general practitioner (GP) respondents and non-respondents in terms of age and gender.

**Variable**	**All GPs** **(n = 80)**	**Respondents** **(n = 56)**	**Non-respondents** **(n = 24)**	**Test value** **[p value]**
**Age**				

Mean age (SD)	50.5 (8.1)	49.9 (7.8)	52.1 (8.7)	t = -1.122 [p = 0.265]

**Gender**				

Male	55 (69)	42 (75)	13 (54)	X^2 ^= 3.3.94 [p = 0.065]

Female	25 (31)	14 (25)	11 (46)	

Figures are numbers followed by percentage (%) unless otherwise stated.

### Respondents

The mean age (SD) of respondent general practitioners was 49.9 (7.8) years, 73% (40/56) were male and 55% (30/56) worked in single handed practice. The average list size was 591 patients with a range of 20–1597. The mean number of refugee and asylum seeker patients per list was 18, with a range of 1–196.

### Need for language assistance

**Figure 1:**
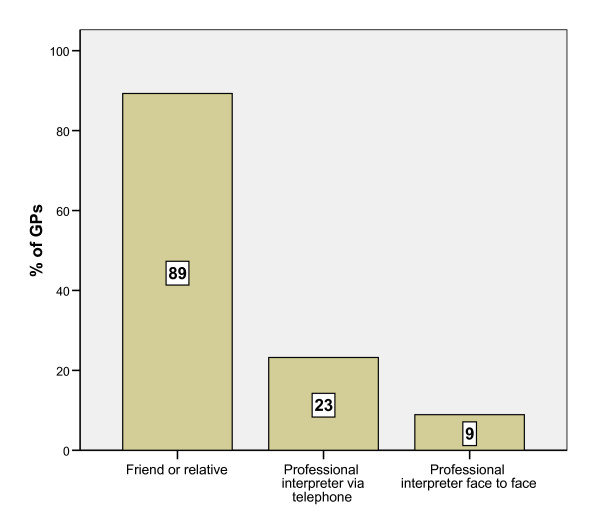
**Forms of interpretation used by GPs during consultations with refugees and asylum seekers where language assistance required**.

The majority of respondents (77%) had experienced consultations with refugee and asylum seekers where language assistance was required. Of these, 89% used some form of interpretation during those consultations. Figure 1 illustrates the frequency with which different forms of interpretation were being used by general practitioners during consultations with refugees and asylum seekers where language assistance was required.

At the same time, the majority of respondents (63%) had been in situations where they felt they required an interpreter but they managed without one. In such consultations, communication took place by a variety of methods such as:

• using sign language and diagrams (46%)

• patient was able to speak some English (18%)

• general practitioners having some knowledge of the patient's language (32%)

Sometimes, a combination of these methods was used in a consultation.

### Knowledge and use of professional interpreters

As little as 7% of respondents could name a professional interpreting agency while only 5% could name a professional interpreting agency that they had actually used. Only 37% of respondents were aware that the HSE WA had funds to support the use of professional interpreters during consultations. When given the choice, 48% of respondents said they had a preference for working with a face to face professional interpreter because they felt it was more effective and satisfactory, while 3% had a preference for a telephone professional interpreter because it was quicker and more practical. The remainder of respondents did not express a preference.

### Use of informal interpreters

Over half (56%) of respondents felt that the primary advantage of working with an informal interpreter was that the patient and interpreter know each other. 18% cited availability as the primary advantage and 8% said that it meant that communication could take place there and then. Possible disadvantages given by respondents for working with informal interpreters were that it posed confidentiality issues (43%), there was a risk of mis-interpretation by the informal interpreter (9%) as well as a recognised lack of medical terminology (11%).

### Professional vs informaI interpreters

**Figure 2:**
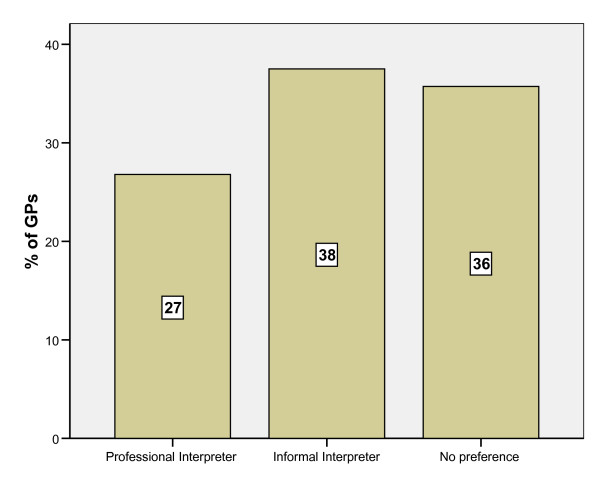
**Preference for type of interpretation used in consultations with refugees and asylum seekers where language assistance required**.

When given the choice of working with a professional, or informal interpreter, a greater number of respondents said they had a preference for working with an informal interpreter (the primary reason cited being accessibility) than with a professional interpreter (the primary reason cited being accuracy) [Figure 2]. However, confidentiality was seen as a greater problem in informal interpretation (43%) than in the professional setting (11%).

## Discussion

### Language assistance: need and responses

The need for language assistance in consultations with refugees and asylum seekers is high at 77%. The experience of the language barrier in Irish general practice is, in fact, likely to be larger if figures from populations of migrant workers and not only refugees were to be included. This emphasises the importance for future research in this area that is inclusive of all communities who may have limited English.

This study indicates that Irish general practitioners are responding to the need for language assistance in a variety of ways including the use of professional interpreters, informal interpreters and 'getting by' with sign language, gestures or limited amounts of English or the patient's language (e.g. French for French speaking African patients). Of note here, is the high proportion of general practitioners who 'get by' without any form of interpretation (professional or informal). There is no empirical data on the efficacy of these strategies but it is unlikely that they facilitate accurate and comprehensive information exchange and communication.

We have also identified that, individual general practitioners respond in different ways at different times showing that they do not have a 'set' response to language barriers. This concurs with previous international studies that use of professional interpreters in Ireland is *ad hoc *and not consistent [15-20]

Levels of knowledge about professional interpreters were very low and just over a third of the sample knew that the HSE WA provided funds for the costs of professional interpreters. These low levels of knowledge are reflected in low levels of use – less than a fifth of the sample reported use of professional interpreters. Improving general practitioners' knowledge of available professional interpreting services is obviously important. There are three points to make here. First, as previously mentioned, professional interpreters in Ireland are not always trained. Therefore, questions remain about promoting the use of untrained interpreters.

Second, increased knowledge about available professional interpretation services in Ireland or other countries is not likely to be sufficient to improve uptake. Recent research highlights that practice characteristics influence uptake of professional interpreters thus emphasising that *organisational contexts *shape general practitioners' behaviour and responses to language barriers [17]. Future national and international research should attend to a whole systems analysis of language barriers in general practice.

Third, general practitioners in this study do not indicate a strong preference for professional interpreting over informal interpreting. They had views about the advantages and disadvantages of professional and informal interpreting which reveal some of the complexities involved in this area. Professional interpreting was considered to be more accurate but, at the same time, it was considered time consuming and difficult to access. While general practitioners reported concerns about confidentiality in consultations involving informal interpreters, they do sometimes have a preference for working with informal interpreters because of their accessibility.

This indicates that general practitioners do see merits in both 'best practice' (professional interpreting) and 'second best practice' (informal interpreting) [16]. These findings resonate with recent research with general practitioners, patients and interpreters which elucidate the unanticipated merits of different kinds of interpreting for the different parties involved [16,25]. It would be fruitful to create a dialogue between all three parties involved in interpreted consultations (general practitioner, patient and interpreter) about each others' perspectives to (a) generate shared understanding of the relative merits and demerits of the range of responses currently in use and (b) enhance knowledge about the organisational levers and barriers to the uptake of trained, professional interpreters Finally, it is important to think as well about education and training for general practitioners and other healthcare professionals who, increasingly, require skills to work in transnational medical encounters [26]. There are many existing courses and resources that can be drawn on to impact on training health professionals [e.g. [27]]. At the National University of Ireland, Galway, School of Medicine, we are devising a Special Study Option for undergraduate medical students about 'Valuing Diversity' and anticipate evaluation of that in 2008–2009.

### Study limitations

The response rate in this survey was very good, which confirmed our choice of a telephone rather than a postal survey. The questionnaire for the telephone survey was not internally validated however, as stated above, it was developed with reference to international literature and in consultation with experts in refugee and asylum seeker health, language interpretation and primary care.

Data reported here are based on retrospective accounts of accumulative experiences. Prospective studies of individual consultations would provide a more accurate assessment of the language assistance within specific time frames (e.g. a day or week) and the proportion of consultations per day requiring language assistance. Such data would be useful to increase quantitative knowledge about language barriers in general practice. The study was small scale but does provide important quantitative data on a national and international issue for general practice.

## Conclusion

The need for language assistance in consultations with refugees and asylum seekers in Irish general practice is high. General practitioners rely on informal responses. It is necessary to improve knowledge about the organisational contexts that shape general practitioners responses. We also recommend dialogue between general practitioners, patients and interpreters about the relative merits of informal and professional methods of interpretation so that general practitioners' choices are responsive to the needs of patients with limited English.

## Abbreviations

HSE WA: Health Services Executive Western Area

## Competing interests

The authors declare that they have no competing interests.

## Authors' contributions

AMacF is Principal Investigator. She designed and supervised the survey and led the write up of this paper. LG conducted the analysis and drafted the paper. PM collected the data. AWM contributed to study design. PM and AWM commented on drafts of the paper. All authors have read and approved the final draft.

## Pre-publication history

The pre-publication history for this paper can be accessed here:



## Supplementary Material

Additional file 1 Summary of Questions in Telephone Survey. A summary of questions used to gather data during the telephone survey with general practitioners. Click here for file
