# Supplementary material for: Responses to language barriers in consultations with refugees and asylum seekers: a telephone survey of Irish general practitioners
Source: BMC Fam Pract. 2008 Dec 22;9:68. doi: 10.1186/1471-2296-9-68 (PMC2637872; doi:10.1186/1471-2296-9-68)
Supplement: Additional file 1 — Summary of Questions in Telephone Survey. A summary of questions used to gather data during the telephone survey with general practitioners. [file 1471-2296-9-68-S1.doc]

**Box 1 Summary of Questions in Telephone Survey**

Specifics of Questions Asked:

Questions determined whether some of their refugee and asylum seeking patients required language assistance and whether professional or informal interpreters were used.

Practitioners knowledge (names of services, sources of information, information about payment for services) of professional services

Practitioners use (access and quality of interpretation) of professional language interpretation services.

Views about the primary advantages and disadvantages of professional services

Practitioners views about primary advantage and disadvantage of informal interpreters

Views about the quality of informal interpreters

Preferences for professional or informal interpreters
